# Ocular toxocariasis presenting as bilateral scleritis with suspect retinal granuloma in the nerve fiber layer: a case report

**DOI:** 10.1186/s12879-016-1762-1

**Published:** 2016-08-18

**Authors:** Kang Yeun Pak, Sung Who Park, Ik Soo Byon, Ji Eun Lee

**Affiliations:** 1 Department of Ophthalmology, Haeundae Paik Hospital, Inje University, Busan, Korea; 2 Department of Ophthalmology, School of Medicine, Pusan National University, Yangsan, Korea; 3 Biomedical Research Institute, Pusan National University Hospital, Busan, Korea; 4 Research Institute for Convergence of Biomedical Science and Technology, Pusan National University Yangsan Hospital, Yangsan, Korea

**Keywords:** Ocular toxocariasis, Scleritis, Toxocara, Visceral larva migrans, Retinal granuloma

## Abstract

**Background:**

This report details ocular toxocariasis presenting as bilateral scleritis with suspect retinal granuloma in the nerve fiber layer.

**Case presentation:**

The patient presented with scleritis, which did not improve with systemic steroid. Intraocular pressure was elevated, and well demarcated hyper-reflective round lesion were noted in both eyes. He had a history of general ache and concurrent onset of ocular symptoms the day after eating raw meat. Systemic work-ups revealed no remarkable abnormalities except antibody for toxocara. Oral albendazole and steroid were prescribed. The inflammation and swellings resolved without recurrence. In the current case, scleritis with suspect granuloma in the nerve fiber layer seems to be caused by toxocara.

**Conclusion:**

Ocular toxocariasis can be presented as atypical features. Serologic exams for toxocariasis would be considered not only in typical features but also in other uveitis or scleritis, particularly when the patient has a related history.

## Background

Scleritis is an ocular inflammatory disorder often associated with ocular or systemic diseases [1]. Although the majority of cases are autoimmune in origin, infectious diseases are potential causes of scleritis [2]. Herpes virus is the most common cause of scleritis associated with infection [2], and other organisms were also reported [3–7]. However, to the best of our knowledge, there has been no report of scleritis associated with ocular toxocariasis (OT).

Although OT is usually diagnosed clinically by identifying typical signs of retinal granuloma or nematode endophthalmitis [8], atypical presentations without granuloma such as invasion of ciliary body [9] or lens [10] and optic nerve swelling [11] have also been reported. A number of OT may be under-diagnosed due to the limitations of diagnostic tools. Here, we report a case of OT presenting as bilateral scleritis with suspect retinal granuloma in the nerve fiber layer.

## Case presentation

A 68-year old male presented with ocular pain and redness for 4 weeks. He ingested raw meat about 1 month before, and ocular symptoms developed with general ache the next day. The patient didn’t have history related with pets. He frequently had eaten the uncooked meat.

He had been treated with topical and systemic steroid in another clinic for 2 weeks, and was referred to our clinic due to uncontrolled inflammation and intraocular pressure (IOP). Medical history and systemic work-ups for conditions related to scleritis, including herpes virus, Wegener’s granulomatosis, rheumatoid arthritis and inflammatory bowel diseases, revealed no remarkable abnormality.

**Fig. 1:**
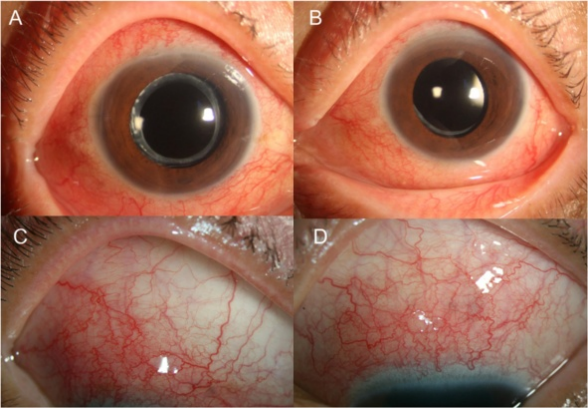
Anterior segment of the right (**a** and **c**) and left eye (**b** and **d**). **(a)** and (**b**) show diffuse injections of both eyes. Episcleral and deep scleral vessels were engorged diffusely (**c** and **d**)

**Fig. 2:**
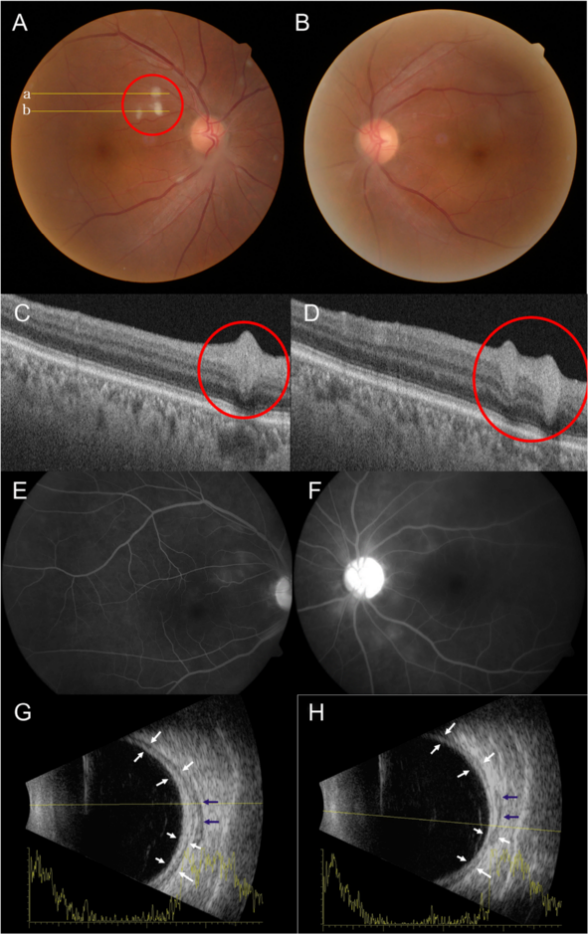
Suspect retinal granulomas (*red circles*) of the right eye at presentation. **a** Fundus photography showed three whitish plaques (*red circle*) on the right eye, mimicking cotton wool spots. **b** There was no lesion in the left eye. **c** Optical coherent tomography (OCT) scan, corresponding to line ‘a’, shows a well demarcated oval shape lesions without shadowing in retinal nerve fiber swelling (*red circle*). **d** OCT scan corresponding to line ‘**b**’ demonstrates two lesions (*red circle*). **e** and **f** Fluorescein angiography showing mild leakage around the optic disc in the late phase. Ultrasonography of the right eye (**g**) and left eye (**h**). White arrows indicate thickened sclera and black arrows point to fluid collection

**Fig. 3:**
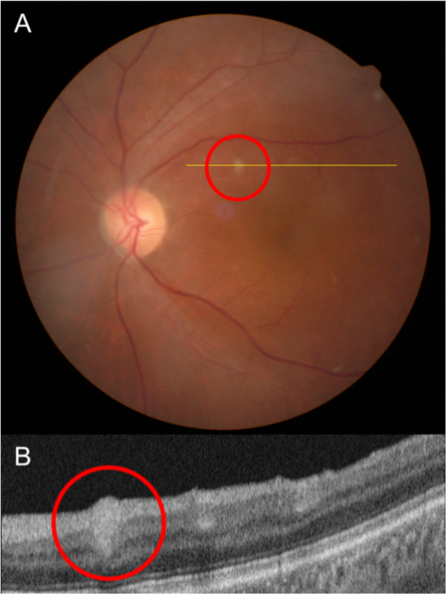
Clinical presentation after 3 weeks. **a** Suspect retinal granuloma in the retinal nerve fiber (*red circles*) developed in the left eye. Fundus photography shows a new whitish plaque (*red circle*) in the left eye. **b** Optical coherent tomography scan corresponding to the line shows that the lesions located in inner retina and have well-demarcated margin without posterior shadowing (*red circle*)

He had been using topical steroid and IOP-lowering drugs and taking oral steroid (prednisolone 15 mg/days). The best corrected visual acuity (BCVA) was 20/20 in both eyes, and IOP was 35 mmHg in the right eye and 36 mmHg in the left eye. He was pseudophakic in both eyes. The episcleral and deep scleral vessels were engorged diffusely, and 0.5+ cells were noted in the anterior chamber of both eyes (Fig. 1). There was no remarkable sign in visual field test or gonioscopy. Vitreous haziness was not detected. Three whitish plaques mimicking cotton wool spots were found in the para-foveal area of the right eye. Optical coherent tomography (OCT) depicted well demarcated hyper-reflective round lesion in the retinal nerve fibers (RNF) layer with posterior shadowing (Fig. 2). These findings suggest retinal granuloma rather than infarction in the RNF. Ultrasonography showed diffuse thickening of the sclera in both eyes. Fluorescein angiography demonstrated no abnormal hyper-fluorescein in the early phase and mild leakage around optic disc and whitish spots in the late phase (Fig. 2). A white spot similar to the lesions developed in the left eye 10 days later (Fig. 3).

**Fig. 4:**
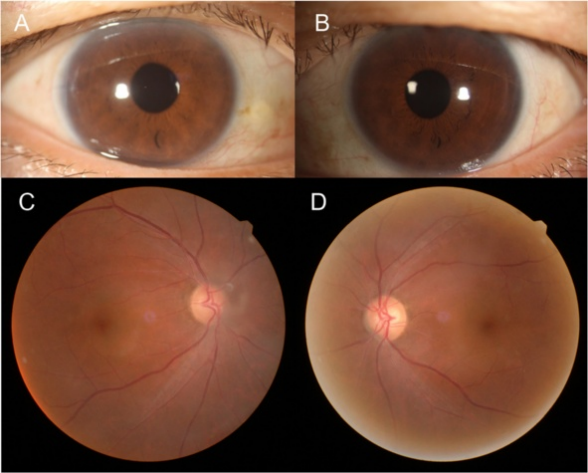
Clinical presentation after 5 months. Anterior segment photos of the right eye (**a**) and left eye (**b**) show no inflammatory signs. Fundus photo of the right eye (**c**) and left eye (**d**) demonstrates that the multiple whitish lesions disappeared

Considering that his symptoms presented just after eating raw meat, additional laboratory work-ups for parasites were performed. The serologic evaluation detected specific immunoglobulin G antibody against toxocara, but no other organisms including Cysticercus, Paragonimus, Sparganum, and Clonorchis. Albendazole (400 mg bid/day) was prescribed for 10 days, combined with oral prednisolone (30 mg/day). The scleritis resolved and IOP became normal by 2 weeks. All medications were discontinued at 3 weeks. The whitish lesions also disappeared (Fig. 4). There was no recurrence until 5 months after stopping medications.

## Discussion

The majority of underlying diseases of scleritis are autoimmune in origin. However, it has been also reported that various infectious organisms, including virus, bacteria, fungus, or protozoa, may cause scleritis [2–7]. Although the remaining cases are classified as idiopathic, it is supposed that an idiopathic disease can be found to have associations with a specific disease in future. This is important to conduct a disease-specific treatment, which would be more effective with fewer side effects.

Although most OT was thought to develop in pediatric patients [8], recent reports indicated that adult patients were predominantly affected by OT especially in Asian populations where ingestion of unheated meat is not infrequent [12]. Systemic symptoms of toxocariasis can appear as mild discomfort, classical visceral larva migrants presenting with severe general illness, or asymptomatic [13]. Conversely, ocular symptoms of OT vary depending on the primary site involved and the immune response of the host [1]. Chorioretinal granuloma with uveitis is considered a typical finding of OT [8]. Although a definitive diagnosis for OT is made histologically by identifying the toxocara larva from a biopsy [8], it can be diagnosed clinically based on typical ocular findings in order to avoid the risks of biopsy [8]. Additionally, laboratory work-ups such as enzyme linked immunosorbent assay (ELISA) or and eosinophilia play an auxiliary role in diagnosis [14]. Although systemic eosinophilia is an important feature of systemic Toxocariasis, eosinophilia count is not usually elevated in OT patients [8].

OT can be easily misdiagnosed, when typical granuloma in the chorioretina is not presented. Several reports have indicated that toxocara can involve the ciliary body [9], lens [10], or optic nerve without granuloma [14], and it is challenging to diagnose OT in these cases.

In the present case, it was assumed that the patient ingested toxocara by eating unheated meat. The next-day myalgia represented visceral migrans syndrome. When he visited the clinic for the first time for his ocular symptoms, the general illness had already been resolved; hence, it was difficult to find an association between ocular inflammation and his systemic symptoms.

The whitish lesions we observed in the patient were interesting. The whitish plaques were in the superficial retina (Figs. 3 and 4) mimicking cotton wool spot. However, OCT depicted well demarcated oval shaped lesion in the RNF layer with posterior shadowing. The size of these plaques was about 100 to 150 μm in diameter, and larger than toxocara larvae or eggs. Although these lesions are different from typical granuloma that shows irregular margin in OCT [11, 12], they resolved after anti-toxocara medication, and appear to be granuloma caused by toxocara accompanying less inflammatory reaction.

As there was no report to compare the superiority of anthelminthic drug in OT, the standard treatment of anti-toxocariasis was administered using albendazole and systemic steroid following the previous report [8, 15, 16], and both retinal granuloma and scleritis were resolved successfully.

## Conclusions

A patient with history of eating unheated meal presented with bilateral scleritis and retinal granuloma. Specific past history, positive serologic tests for toxocara, and treatment responses suggested that scleritis were manifestations of OT. Scleritis should be considered as one of manifestation of OT, and was managed with the standard anti-toxocariasis medication. Serologic exams for toxocariasis would be considered not only in typical features but also in other uveitis or scleritis, particularly when the patient has a related history.

## Abbreviation

BCVA, best corrected visual acuity; IOP, intraocular pressure; OCT, optical coherent tomography; OT, ocular toxocariasis; RNF, retinal nerve fiber
